# Anti-neuroinflammatory effects of conjugated linoleic acid isomers, c9,t11 and t10,c12, on activated BV-2 microglial cells

**DOI:** 10.3389/fncel.2024.1442786

**Published:** 2024-09-27

**Authors:** Clara Porcedda, Claudia Manca, Gianfranca Carta, Franca Piras, Sebastiano Banni, Valeria Sogos, Elisabetta Murru

**Affiliations:** Department of Biomedical Sciences, University of Cagliari, Monserrato, Italy

**Keywords:** conjugated linoleic acid (CLA), microglia, neuroinflammation, *N*-acylethanolamines (NAEs), inflammatory mediators

## Abstract

Conjugated linoleic acid (CLA) isomers exhibit anti-inflammatory properties within the central nervous system (CNS). This study investigated the effects of CLA isomers c9,t11 and t10,c12 on fatty acid (FA) and *N-*acylethanolamine (NAE) profiles and their association with pro-inflammatory molecule expression in BV-2 microglia cell line, the CNS's resident immune cells responsible for maintaining neuronal activity and immune homeostasis. BV-2 cells were treated with 25 μM of c9,t11-CLA, t10,c12-CLA, or oleic acid (OA) for 24 h, followed by lipopolysaccharide (LPS) stimulation. After treatment, the cell's FA and NAE profiles and pro-inflammatory molecule expression were analyzed. Our results demonstrated that CLA isomers mitigate LPS-induced morphological changes in BV-2 cells and reduce gene expression and protein levels of inflammatory markers. This effect was linked to an upregulation of acyl-CoA oxidase 1, a key enzyme in the anti-inflammatory peroxisomal beta-oxidation pathway that efficiently metabolizes CLA isomers. Notably, t10,c12-CLA significantly suppressed stearoyl-CoA desaturase 1, impacting monounsaturated fatty acid synthesis. The NAEs profile was remarkably altered by CLA isomers, with a significant release of the anti-neuroinflammatory mediator docosahexaenoic acid (DHA)-derived *N-*acylethanolamine (DHAEA). In conclusion, our findings suggest that the anti-neuroinflammatory effects of CLA isomers are due to their unique influences on FA metabolism and the modulation of bioactive FA-derived NAEs, highlighting a potential strategy for nutritional intervention in conditions characterized by neuroinflammation.

## 1 Introduction

Microglia represent the resident immune cells in the central nervous system (CNS) that actively survey the microenvironment and ensure normal neuronal activity and immunological integrity (Muzio et al., 2021). These cells protect the brain by phagocytosing bacteria, cellular debris, protein aggregates, and other antigens that may pose a threat to the CNS. Beyond their protective role, microglia are instrumental in neural network remodeling, synaptic pruning, and neurogenesis (Wake et al., 2011). Under pathological conditions, microglia transition to an activated state, adopting an amoeboid form and secreting inflammatory molecules like cytokines, chemokines, eicosanoids, reactive oxygen species, and nitric oxide, targeting pathogens or infected cells (Colonna and Butovsky, 2017). However, chronic or excessive activation can lead to detrimental inflammation, contributing to neurodegenerative diseases' progression by causing neuronal loss. Notably, neuroinflammation is a critical factor in various brain disorders, including ischemic events and neurodegenerative diseases like Alzheimer's, Parkinson's, and multiple sclerosis (Wendimu and Hooks, 2022). In aging, microglia activation is linked to increased sensitivity, potentially accelerating age-related neurodegeneration and disrupting microglia-synapse interactions (Du et al., 2017). Thus, controlling microglial activation might mitigate neuronal impairment in neurological conditions and during aging (Angelova and Brown, 2019).

Dietary fatty acids (FAs) are able to cross the blood-brain barrier (BBB) and are mostly esterified into membrane phospholipids of neurons and glial cells. These FAs influence brain functions directly or through their active metabolites (Bazinet and Layé, 2014). Recent findings suggest FAs can either promote or inhibit microglial inflammatory responses (Leyrolle et al., 2019). Specifically, saturated fatty acids (SFAs) trigger a pro-inflammatory microglial phenotype associated with neurodegeneration (Wang et al., 2012), while n-3 polyunsaturated FAs elicit anti-inflammatory effects, reducing apoptosis in the rodent brain (Jia et al., 2014).

Previous research has shown that conjugated linoleic acid (CLA) isomers, prevalent in meat and dairy, are actively utilized and metabolized in rat brains and cultured astrocytes, influencing the inflammatory response (Fa et al., 2005; Saba et al., 2019). Particularly, a major metabolite from CLA metabolism was identified as CD16:2, derived from its partial peroxisomal β-oxidation, hinting at a specific peroxisomal metabolism pathway for CLA (Fa et al., 2005). Among the isomers, c9,t11-CLA is predominant in dietary sources (Wahle et al., 2004) and has been linked to beneficial effects on neural cells and potential protection against neurodegenerative diseases (Fujita et al., 2021). Specifically, *in vitro* studies have shown that c9,t11-CLA promotes the proliferation of neural progenitor cells (Wang et al., 2011) and protects from glutamate- or Aβ-induced neuronal cell death (Hunt et al., 2010; Lee et al., 2013).

CLA not only affects FAs metabolism but also impacts the biosynthesis of *N-*acylethanolamines (NAEs), part of the endocannabinoidome, which regulates neuroprotection and inflammation (Batetta et al., 2009; Di Marzo et al., 2010; Murru et al., 2021a).

The interplay between CLA isomers and microglial activation, particularly through the NF-κB pathway, suggests a neuroprotective role against diet-induced brain inflammation (Salsinha et al., 2023).

The present study aims to assess the anti-inflammatory potential of CLA isomers on microglial cells *in vitro*, focusing on their impact on FAs metabolism, lipid mediator synthesis, and inflammatory molecule production. The findings could provide new insights into dietary CLA's role in mitigating neuroinflammation and its relevance to neurodegenerative diseases.

## 2 Materials and methods

### 2.1 Cell line and culture conditions

**Figure 1:**
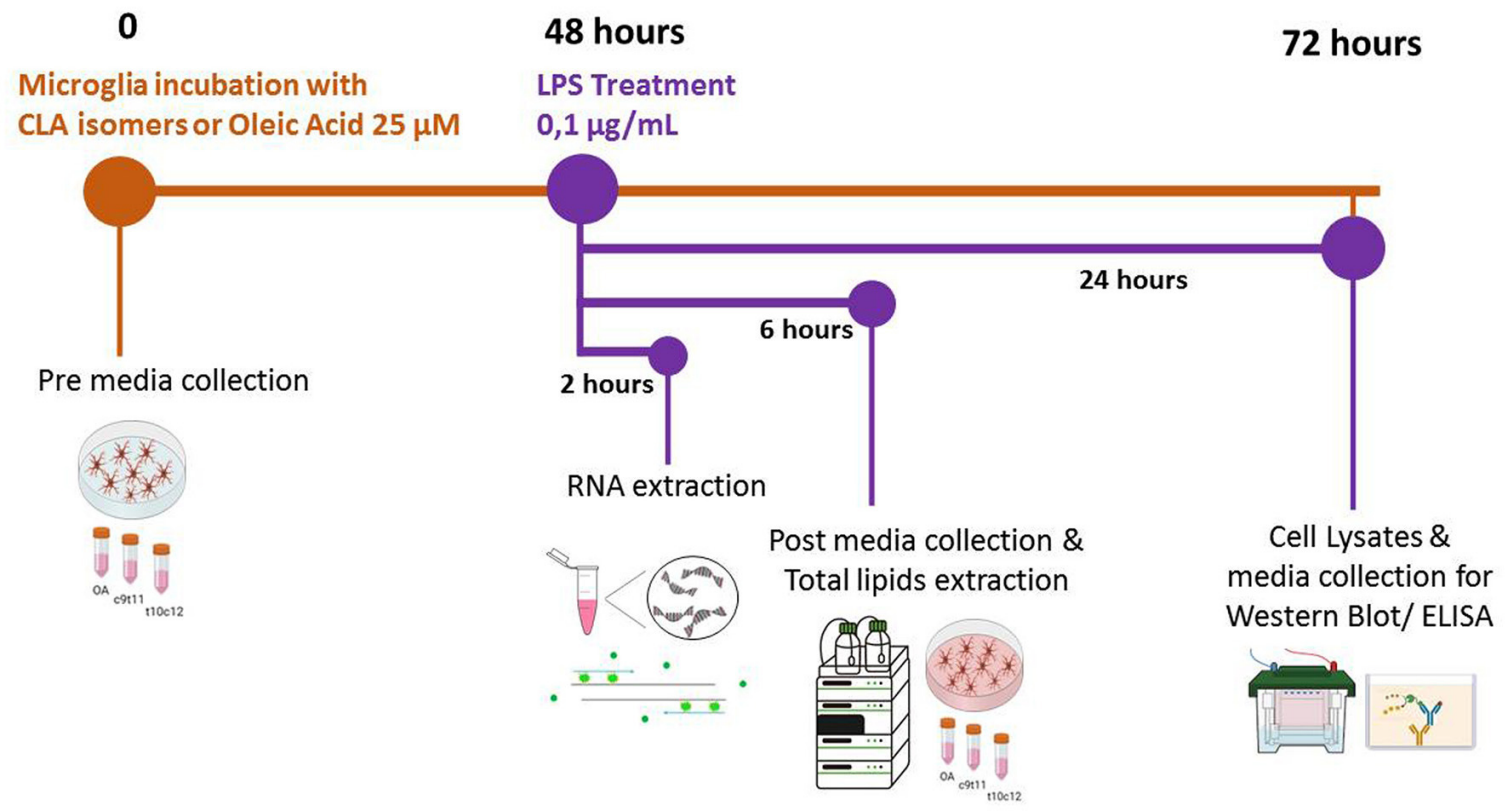
Timeline of the experimental procedures. At the initial time point (0 h), cells were incubated in complete culture media supplemented with 25 μM of either c9,t11-conjugated linoleic acid (CLA), t10,c12-CLA, or oleic acid (OA). Aliquots of this culture medium, henceforth referred to as “pre media”, were collected for subsequent analysis. After a 48-h incubation period, microglia were stimulated with lipopolysaccharide (LPS) for 2, 6, or 24 h, contingent upon the specific analyses planned. Following 6 h of LPS stimulation, additional media samples, designated as “post media”, were collected to enable a comparative analysis of fatty acid (FA) release relative to the “pre media”.

BV-2 microglial cells were cultured (2 × 10^4^cells/ml) in complete medium containing low glucose DMEM supplemented with 10% fetal bovine serum (FBS), 1% penicillin/streptomycin (all from Gibco, Thermo Fisher Scientific), and maintained at 37°C with an atmosphere of 5% CO_2_. After 24 h, the medium was changed, and cells were incubated with 25 μM of c9,t11-CLA (#16413, Sigma Aldrich, Merck, Darmstadt, German) or t10,c12-CLA (#90154, Cayman Chemical, Ann Arbor, MI, USA) or oleic acid (OA) (# O-7008, Sigma Aldrich, Merck, Darmstadt, German) in complete medium. OA was used as control FA due to its similar incorporation with CLA into lipid fractions (Banni et al., 2001). Extensive testing confirmed that it does not induce any inflammatory effect on BV2 cells (Supplementary Figure S1). To establish a cellular model of neuroinflammation, after 48 h of administration of FAs, cells were exposed to lipopolysaccharide (LPS) 0.1 μg/ml (*E. coli* O26:B6, Sigma Aldrich, Merck, Darmstadt, German) and collected for mRNA, lipids and protein analysis as described in Figure 1.

### 2.2 Real-time PCR

After 2 h of LPS treatment, total RNA was extracted using TRI Reagent (Sigma Aldrich, Merck, Darmstadt, German) according to the manufacturer's instructions. Total RNA was reversely transcribed into cDNA using the High-Capacity cDNA Reverse Transcription Kit (Applied Biosystems, Thermo Fisher Scientific). Real-time PCR was performed using PowerUp SYBR Green Master Mix (Applied Biosystems, Thermo Fisher Scientific) on Rotor-Gene Q (Qiagen) according to the manufacturer's procedure. The 2^−ΔΔCT^ method was used for relative quantitative calculation, and *TATA-binding protein* (*TBP*) was used as the internal reference gene for normalization. The following specific primers were used for real-time PCR: *Acox1* #qMmuCID0016828 (Bio-Rad, Hercules, California, U.S.A); *Ccl5* #Mm.PT.58.43548565 (IDT, Coralville, IA, USA), *Il-6* #Mm.PT.58.10005566 (IDT, Coralville, IA, USA); *Il-1*β #Mm.PT.58.41616450 (IDT, Coralville, IA, USA); *Inos* #qMmuCI0023087 (Bio-Rad, Hercules, California, U.S.A); *Tbp* #Mm.PT.39a.22214839 (IDT, Coralville, IA, USA).

### 2.3 Enzyme-linked immunosorbent assay

To verify cytokines release, 24 h after LPS treatment, the post medium of BV-2 cell culture was collected, centrifugated and tested with the following commercial kits: interleukin (IL)-6 (Invitrogen, Thermo Fisher Scientific, #88-7064-22), CCL5 also known as RANTES (regulated on activation, normal T cell expressed and secreted) (Invitrogen, Thermo Fisher Scientific, #88-56009-22). The assays were performed following the instructions provided by the manufacturers. The absorbance of the samples was measured at 450 nm using a microplate reader (Chameleon, Hidex).

### 2.4 Western blot

After 24 h of LPS exposure, BV-2 cells were lysed in 2% of sodium dodecyl sulfate (SDS), sonicated and centrifuged at 10,000 × g. Protein concentration was quantified using the Pierce BCA Protein Assay Kit (Sigma Aldrich, Merck, Darmstadt, German). Reducing loading buffer was added and the samples were denatured by boiling for 3 min. Fifteen micrograms of proteins were run on SDS/polyacrylamide gels and transferred onto (PVDF) membranes (Hybond-P, Amersham, Marlborough, MA, USA). Membranes were blocked with 5% of non-fat dry milk for 1 h at room temperature and incubated at 4°C overnight with the primary antibodies anti IL-1β (1:250, ab9722, Abcam, Cambridge, UK) or anti iNOS (1:500; ab178945, Abcam, Cambridge, UK). They were washed and incubated with horseradish-peroxidase-conjugated antirabbit IgG (1:10,000; #111-035-003 Jackson ImmunoResearch, Ely, UK) for 1 h at room temperature. Subsequently, bands were developed with the chemiluminescent substrate Lite a Blot Turbo (Euroclone, Pero, MI, Italy) and visualized with ImageQuant LAS-4000 (GE Healthcare, Little Chalfont, UK). Each experimental group's exposure time was optimized to prevent over-saturation and enable accurate densitometric analysis. Membranes were reprobed with anti GAPDH antibody (1:1,000; Mab 374, Millipore, Merck, Darmstadt, German) to normalize the signal of target proteins. Band intensity was analyzed using Image Studio software (LI-COR, Biosciences, Lincoln, NE, USA).

### 2.5 Lipid extraction and *N*-acylethanolamines and FAs analysis

Total lipids were extracted from the pellet containing the BV-2 cell culture after 48 h incubation with CLA isomers or OA and further 6 h of LPS or vehicle treatment, obtained after centrifugation, according to the method of Folch (Folch et al., 1957). Aliquots of the lipid fraction were mildly saponified in order to obtain free FAs for High Performance Liquid Chromatograph (HPLC) using an Agilent 1100 HPLC System (Agilent, Palo Alto, CA, USA) equipped with a diode array detector. A saponified fraction was methylated to obtained FA methyl esters (FAME) to measure SFAs by a Gas-Chromatograph (GC) (Agilent, Model 6890, Palo Alto) equipped with a flame ionization detector (FID) (Murru et al., 2021b).

Deuterated NAEs were added as internal standards to the samples before lipid extraction for quantification by isotope dilution: *N*-arachidonoylethanolamine [2H]^8^AEA, *N*-oleoylethanolamine [2H]^2^OEA, *N*-palmitoylethanolamine [2H]^4^PEA and *N*-stearoylethanolamine [2H]^3^SEA were purchased from Cayman Chemicals (MI, USA). NAEs quantification was carried out by an Agilent 1260 Infinity II UHPLC system (Agilent, Palo Alto) equipped with a mass spectrometry (MS) Agilent Technologies QQQ triple quadrupole 6420 with electrospray ionization (ESI) source, using positive mode (ESI+) (Murru et al., 2021b).

### 2.6 Statistical analysis

Data were analyzed with GraphPad Prism 8.0.1 software (San Diego, CA, USA) using one-way ANOVA for multiple comparisons. The ROUT method and Shapiro-Wilk normality test were employed to identify and remove any outliers and to assess the normal distribution of the data, respectively. *Post-hoc* analyses were performed with Dunnett's multiple comparison test with significance set at ^*^p ≤ 0.05, ^**^p ≤ 0.01, and ^***^p ≤ 0.001, ^****^p ≤ 0.0001. Data are presented as the mean ± SEM of at least three independent experiments.

## 3 Results

### 3.1 CLA isomers prevent LPS-induced morphological modification in BV-2 cells

**Figure 2:**
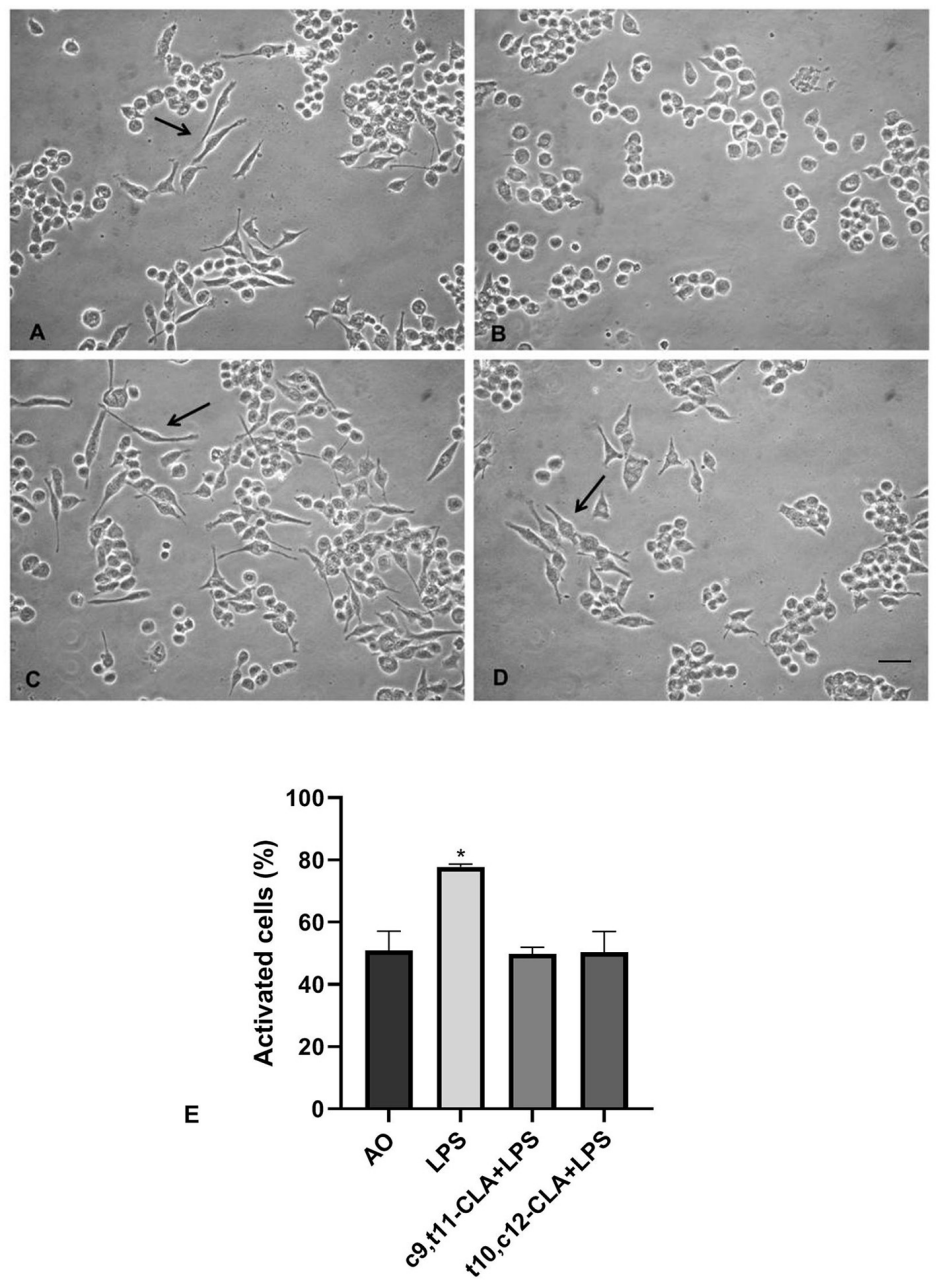
Effects of CLA isomers on LPS-induced morphological changes in BV-2 cells. **(A)** unstimulated BV-2 cells incubated with oleic acid (OA); **(B)** LPS-stimulated BV-2 cells incubated with OA; **(C)** LPS-stimulated BV-2 cells incubated with c9,t11-CLA, or t10,c12-CLA **(D)**. Arrows indicate quiescent morphologies with elongated cellular projections. Bar = 50 μm. **(E)** Quantification of activated BV-2 cells based on their morphological changes. Data represent means ± SEM of *n* = 3 experiments. *p < 0.05 respect to OA.

The control cultures treated with OA (Figure 2A) predominantly exhibited a typical quiescent morphology characterized by elongated cell bodies and cytoplasmic extensions, while the cells subjected to LPS exposure (Figure 2B) transitioned to an activated state, adopting a rounded, amoeboid form. Notably, treatment with either isomer of CLA effectively mitigated the LPS-induced activation of BV-2 cells. Among the isomers, c9,t11-CLA (Figure 2C) demonstrated superior efficacy in preventing the activation compared to t10,c12-CLA (Figures 2D, E).

### 3.2 CLA isomers attenuate gene expression of pro-inflammatory markers

**Figure 3:**
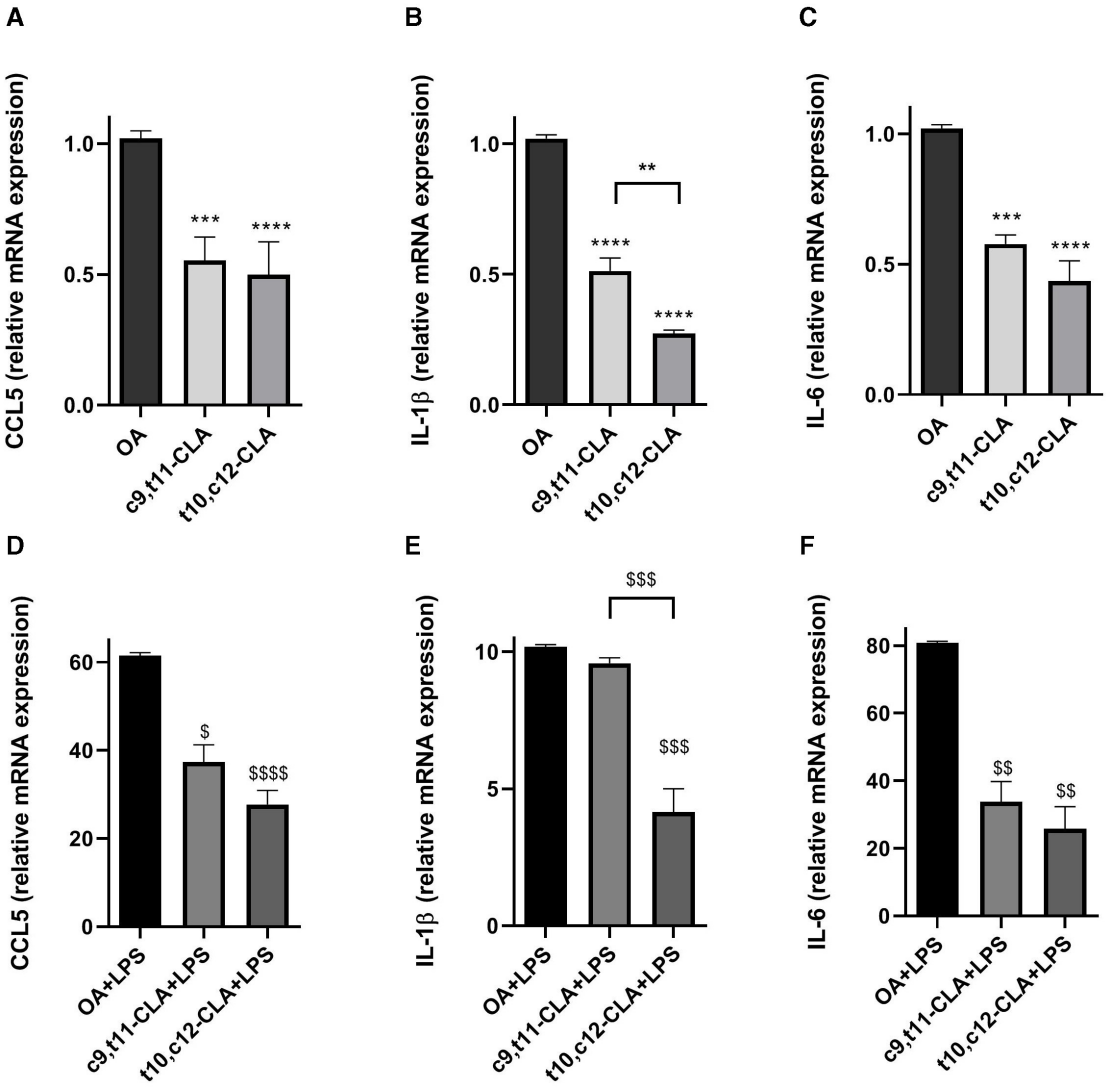
Effects of CLA isomers on the mRNA expression of pro-inflammatory markers CCL5 **(A, D)**, IL-1β **(B, E)** and IL-6 **(C, F)** compared to control (oleic acid, OA), in unstimulated **(A–C)** and LPS-stimulated BV-2 cells **(D–F)**, measured by real-time PCR. The bars represent the fold-change relative to OA. Data represent means ± SEM of *n* = 3 experiments. **p < 0.01, ***p < 0.001, ****p < 0.0001 respect to OA; ^$^*p* < 0.05, ^$$^*p* < 0.01, ^$$$^*p* < 0.001, ^$$$$^*p* < 0.0001 respect to OA + LPS.

To assess the impact of CLA isomers on the mRNA expression of *CCL5, IL-6*, and *IL-1*β, BV-2 microglial cells underwent incubation with c9,t11-CLA or t10,c12-CLA isomers for 48 h, followed by analysis of mRNA levels using real-time PCR. The results, depicted in Figures 3A–C, indicate a notable reduction in the mRNA expression of *CCL5, IL-1*β, and *IL-6* for both CLA isomers. As expected, activation of microglia through LPS exposure markedly increased the mRNA levels of these cytokines. Pre-treatment with c9,t11-CLA significantly mitigated the mRNA expression of *CCL5* and *IL-6* following LPS challenge but did not alter IL-1β levels. Conversely, t10,c12-CLA was effective in significantly reducing the mRNA expression of all three assessed cytokines, as illustrated in Figures 3D–F.

### 3.3 CLA isomers attenuate protein levels of pro-inflammatory markers

**Figure 4:**
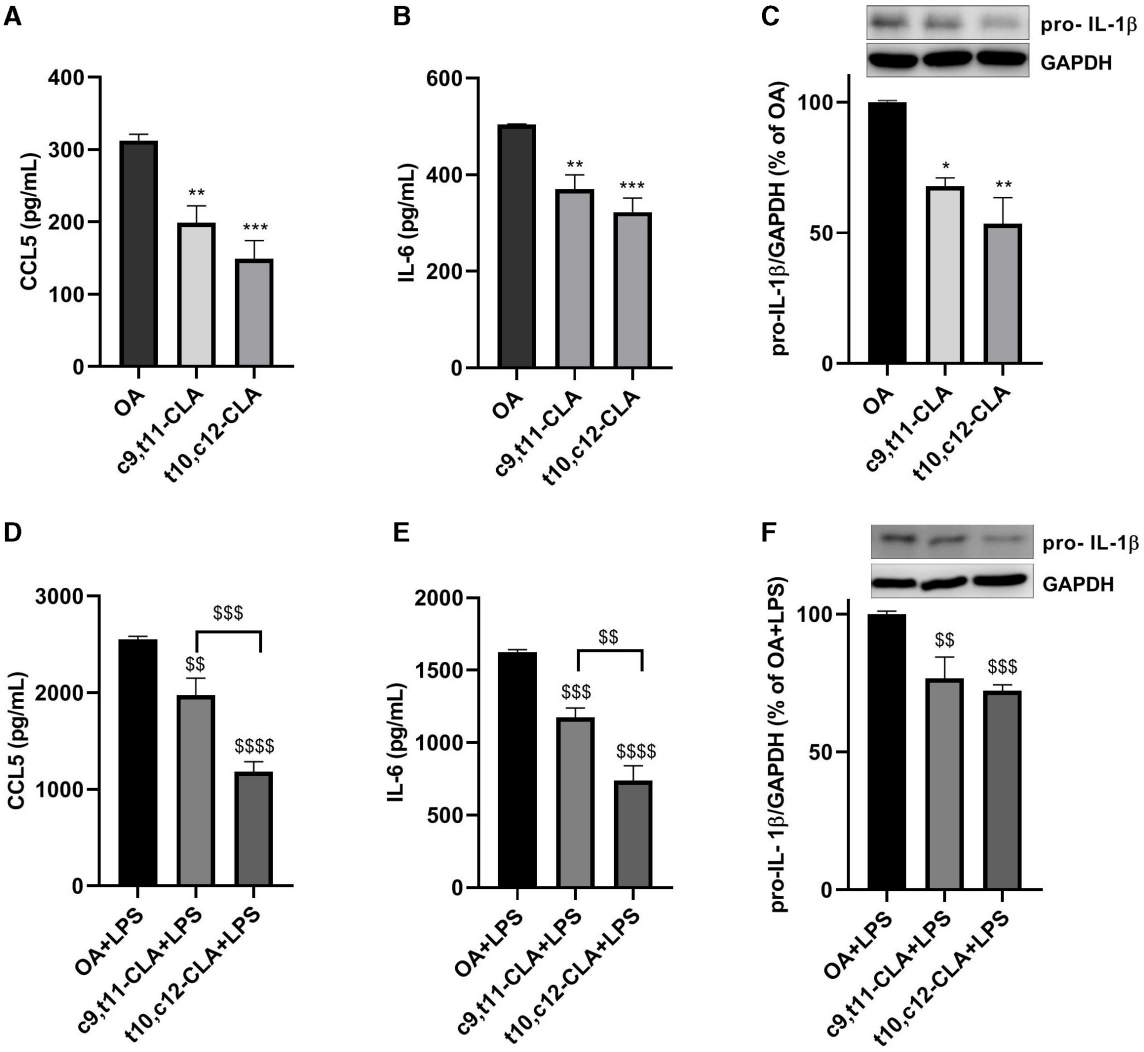
Impact of CLA isomers on the secretion of pro-inflammatory mediators CCL5 **(A, D)**, IL-6 **(B, E)** and pro-IL-1β **(C, F)** in comparison to the control (oleic acid, OA), within the culture media of unstimulated **(A–C)** or LPS-stimulated BV-2 microglial cells **(D–F)**. Data represent means ± SEM of *n* = 3 experiments. *p < 0.05, **p < 0.01, ***p < 0.001 respect to OA; ^$$^*p* < 0.01, ^$$$^*p* < 0.001, ^$$$$^*p* < 0.0001 respect to OA + LPS.

Cytokine secretion was assessed using ELISA in the medium of BV-2 cells treated with CLA isomers. Both c9,t11-CLA and t10,c12-CLA markedly reduced the secretion of CCL5 and IL-6, irrespectively of whether they were exposed to LPS, with t10,c12-CLA demonstrating greater efficacy (Figures 4A, B, D, E). Since IL-1β was not detectable in the medium by ELISA (data not shown), we explored the effects of CLA isomers on IL-1β expression within BV-2 cell extracts. Western blot analysis identified a band at 31 kDa, indicative of the intracellular pro-IL-1β increased expression following LPS induction. Both c9,t11-CLA and t10,c12-CLA notably decreased the expression of pro-IL-1β in cells, regardless of LPS treatment status (Figures 4C–F).

### 3.4 CLA isomers reduce gene expression and production of iNOS

**Figure 5:**
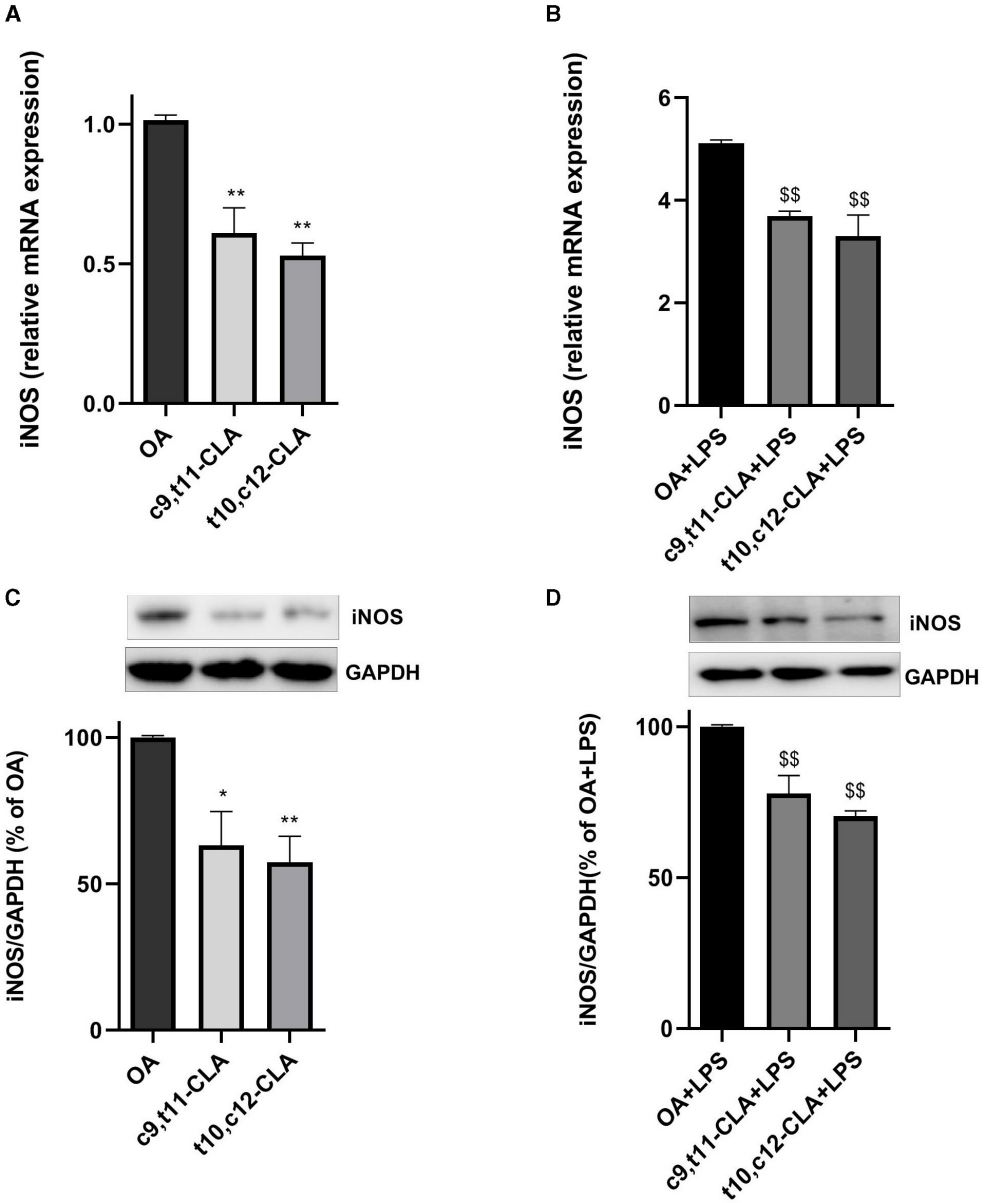
Influence of CLA isomers on iNOS expression in BV-2 cells at both mRNA **(A, B)** and protein **(C, D)** levels, compared to control (oleic acid, OA) in unstimulated and LPS-stimulated BV-2 cells. The results are expressed as fold changes in gene expression relative to the OA-treated group **(A, B)**. The lower panels delineate the effects on iNOS protein levels in unstimulated **(C)** and LPS-stimulated **(D)** BV-2 cells, assessed by Western blot analysis. Data represent means ± SEM of *n* = 3 experiments. *p < 0.05, **p < 0.01 respect to OA; ^$$^*p* < 0.01 respect to OA + LPS.

The inducible nitric oxide synthase (iNOS) is a critical enzyme whose increased levels are indicative of an active inflammatory process. We evaluated the expression levels of iNOS mRNA and protein in BV-2 cells treated with c9,t11-CLA or t10,c12-CLA, followed by a challenge with LPS, using real-time PCR and western blot analysis, respectively. As anticipated, iNOS mRNA and protein expressions were elevated in BV-2 cells following LPS exposure. However, this upregulation was mitigated when cells were pre-treated with either c9,t11-CLA or t10,c12-CLA, with or without LPS challenge (Figure 5).

### 3.5 CLA isomers effect on β-peroxisomal oxidation

**Figure 6:**
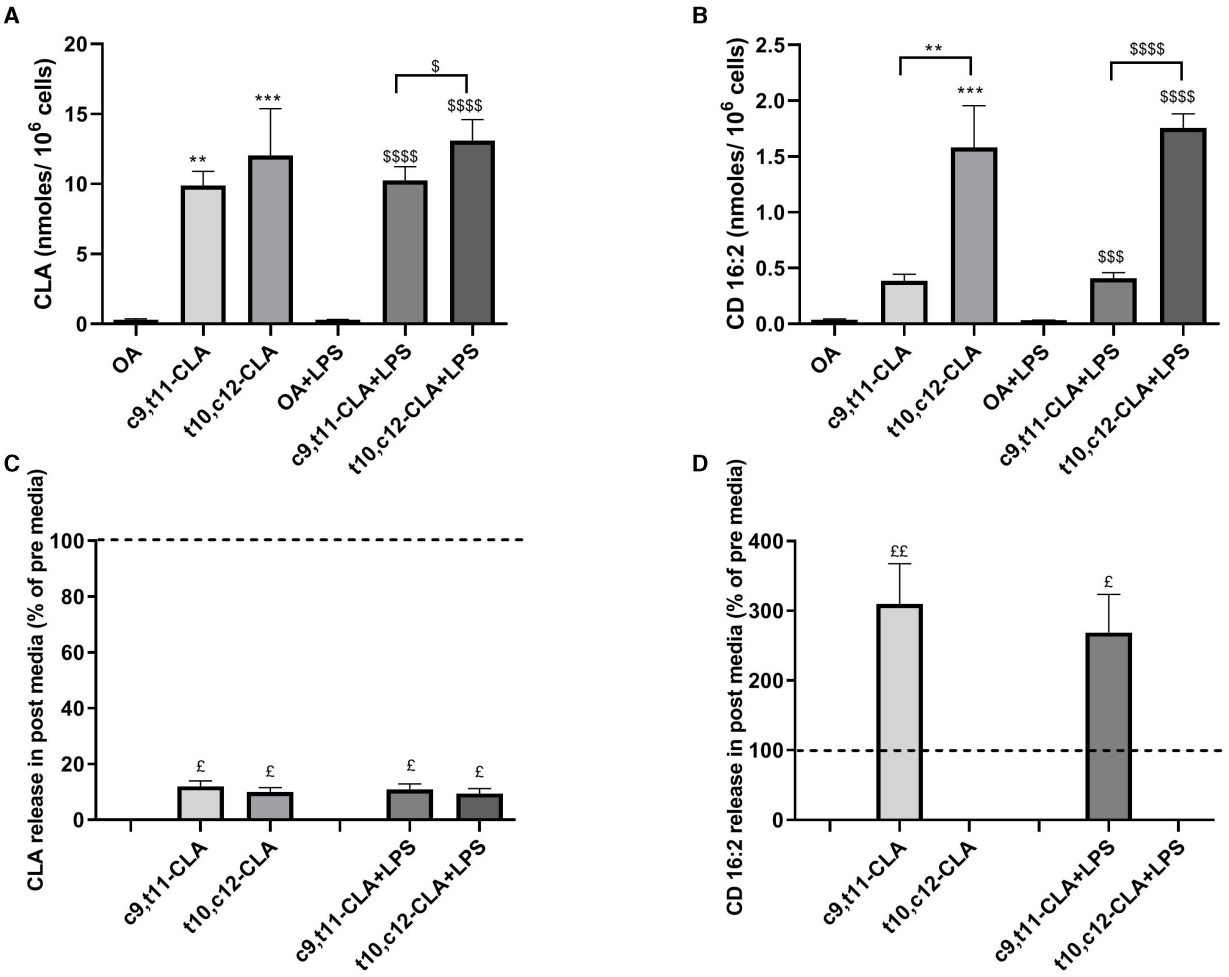
Incorporation of CLA isomers **(A)** and biosynthesis of their peroxisomal β-oxidation product, CD16:2 **(B)** in unstimulated and LPS-stimulated BV2 cells with oleic acid (OA) serving as the control. Data are presented as nmoles/10^6^ cells. Panels **(C, D)** focus on the extracellular release dynamics of CLA and CD16:2, respectively, comparing the concentrations in the media after 48 h of incubation with the isomers, both in the presence and absence of LPS stimulation, relative to initial media concentrations, as indicated by the dashed lines. Data represent means ± SEM of *n* = 3 experiments. **p < 0.01, ***p < 0.001 respect to OA; ^$^*p* < 0.05, ^$$$^*p* < 0.001, ^$$$$^*p* < 0.0001 respect to OA + LPS; ^$^*p* < 0.05, ^$$^*p* < 0.01 respect to pre media.

To determine if the anti-inflammatory effects of CLA isomers are linked to alterations in FAs metabolism, we evaluated changes in the profiles of FAs and bioactive metabolites derived from FAs, such as NAEs, which play a crucial role in modulating inflammatory processes. After 48 h of incubation with CLA, both CLA isomers (c9,t11 and t10,c12) were substantially incorporated into BV-2 cells. After LPS exposure, the assimilation of the t10,c12-CLA isomer was significantly higher by 28% compared to c9,t11-CLA (Figure 6A). Moreover, a marked increase in the concentration of the peroxisomal β-oxidation product CD16:2 was observed for t10,c12-CLA, being roughly three times higher than that from c9,t11-CLA. The accumulation pattern of CD16:2 remained unchanged following LPS treatment (Figure 6B).

The extracellular concentrations of CLA isomers and their metabolites were evaluated by examining the culture media before (pre media) and after treatment (post media). The levels of CLA isomers decreased by ~90% in the media, indicating rapid uptake by the microglia (Figure 6C). Intriguingly, only CD16:2 derived from the c9,t11-CLA isomer was detected in the medium, implying that although both isomers undergo β-oxidation in peroxisomes, c9,t11-CLA is less retained within microglial cells. Moreover, the release of CD16:2 into the medium was marginally reduced by LPS treatment (Figure 6D).

**Figure 7:**
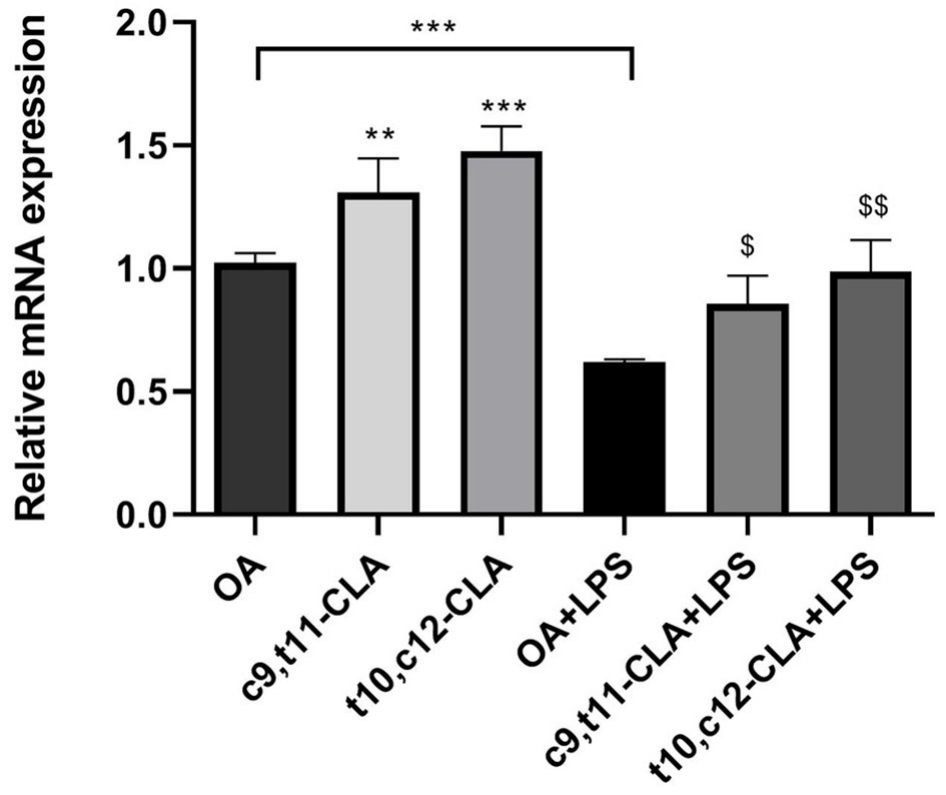
*ACOX1* mRNA expression in both unstimulated and LPS-stimulated BV-2 cells, after a 48-h incubation period with CLA isomers. The bar graph illustrates the fold change in *ACOX1* mRNA expression for each treatment group, normalized to the expression level observed in cells incubated with oleic acid (OA), which serves as the control. Data represent means ± SEM of *n* = 3 experiments. **p < 0.01, ***p < 0.001 respect to OA; ^$^*p* < 0.05, ^$$^*p* < 0.01 respect to OA + LPS.

The effective β-oxidation of CLA in peroxisomes led us to investigate the gene expression levels of acyl-CoA oxidase 1 (*ACOX1*), a pivotal enzyme in this metabolic pathway. Under baseline conditions, treatment with c9,t11-CLA and t10,c12-CLA resulted in elevated *ACOX1* mRNA levels, showing respective increases of ~35 and 45%. However, exposure to LPS reduced *ACOX1* expression by around 45%. Interestingly, the presence of both CLA isomers mitigated the LPS-induced suppression of *ACOX1* expression (Figure 7).

### 3.6 CLA isomers effect on monounsaturated fatty acid metabolism

**Figure 8:**
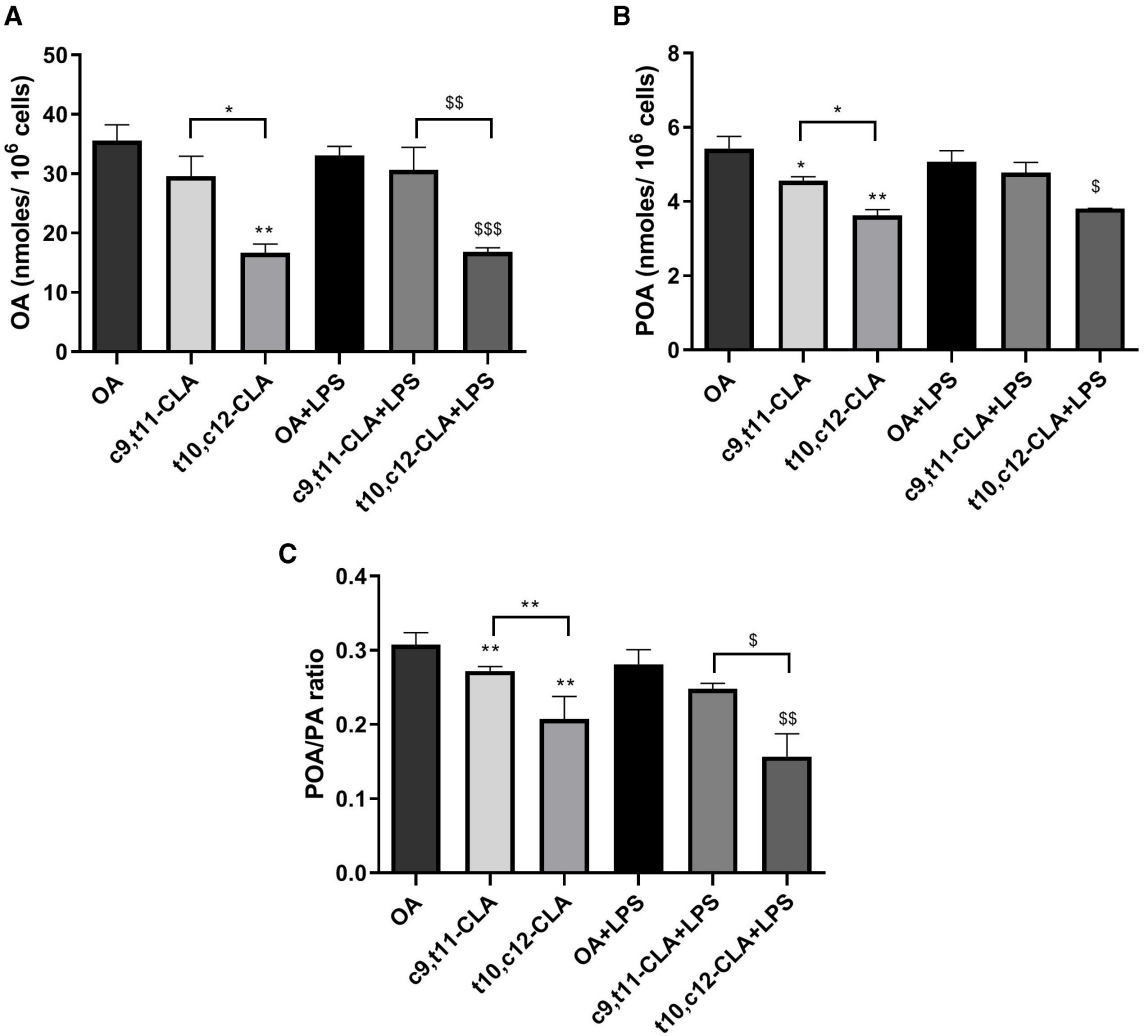
Analysis of main MUFAs oleic acid, OA **(A)**, palmitoleic acid, POA **(B)** and the ratio of palmitoleic acid to its precursor palmitic acid, PA **(C)** in BV-2 cells. These measurements were taken after a 48-h incubation period with c9,t11-CLA or t10,c12-CLA. The data compare these levels in both unstimulated and LPS-stimulated cells to the control group treated with OA. Data represent means ± SEM of *n* = 3 experiments. *p < 0.05, **p < 0.01 respect to OA; ^$^*p* < 0.05, ^$$^*p* < 0.01, ^$$$^*p* < 0.001 respect to OA + LPS.

We conducted analysis of the FAs profiles in BV-2 cells to investigate the potential impact of CLA on cellular lipid metabolism. Treatment with the t10,c12-CLA isomer significantly decreased the levels of OA and palmitoleic acid (POA) when compared to cells incubated with OA or the c9,t11-CLA isomer (Figures 8A, B). The effect of t10,c12-CLA on POA levels was diminished following LPS treatment. Importantly, cells treated with t10,c12-CLA exhibited a reduced POA to palmitic acid (PA) ratio, suggesting a decrease in stearoyl-CoA desaturase-1 (SCD1) activity, which is responsible for the desaturation of SFAs to MUFAs (Figure 8C). No alterations were observed in the concentrations of other FAs.

### 3.7 Impact of CLA isomers on *N-*acylethanolamines biosynthesis

**Figure 9:**
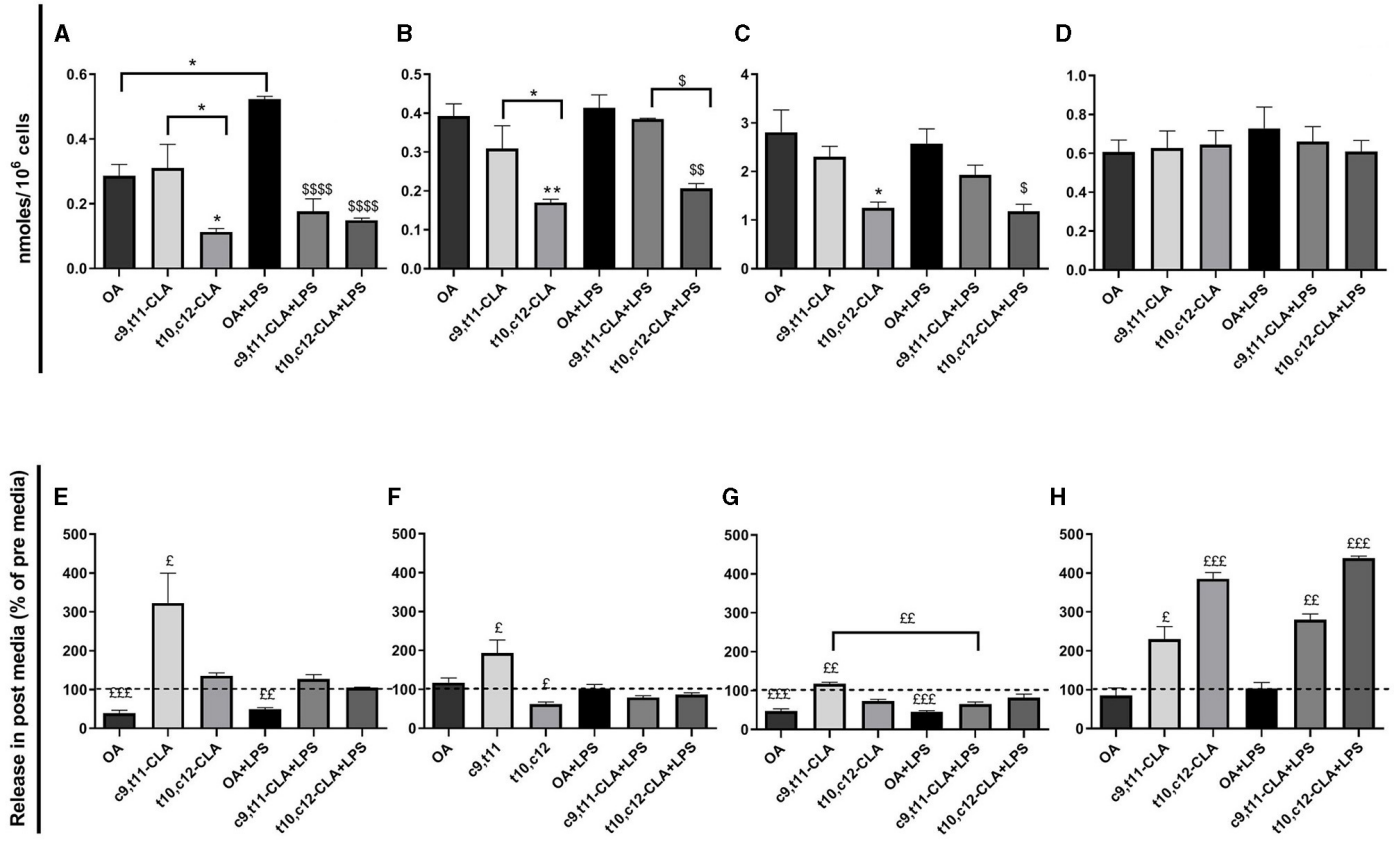
Impact of CLA isomers on the levels of lipid mediators *N*-acylethanolamines (NAEs) within BV2 cells and in media post-incubation (post media): AEA **(A, E)**, POEA **(B, F)**, OEA **(C, G)**, DHAEA **(D, H)**. The intracellular NAEs levels **(A–D)** are quantified in nmoles/106 cells and compared across treatments with the two CLA isomers relative to the OA control. In the post media **(E–H)**, NAEs concentrations are presented as a percentage of their release, with the baseline levels in pre media represented by dashed lines. Data represent means ± SEM of n = 3 experiments. *p < 0.05, **p < 0.01 respect to OA; ^$^*p* < 0.05, ^$$^*p* < 0.01, ^$$$$^*p* < 0.001 respect to OA + LPS; ^$^*p* < 0.05, ^$$^*p* < 0.01, ^$$$^*p* < 0.001 respect to pre media.

Treating BV-2 cells with CLA isomers led to distinct changes in the biosynthesis of lipid mediators, particularly FAs-derived *N-*acylethanolamines (NAEs). Specifically, the reduction in OA and POA levels in cells treated with the t10,c12-CLA isomer was associated with decreased levels of their corresponding NAEs, palmitoleoylethanolamide (POEA) and oleoylethanolamide (OEA) as shown in Figures 9B, C, respectively. Notably, these changes were not influenced by LPS treatment.

Additionally, arachidonoylethanolamide (AEA), a NAE derived from arachidonic acid, was reduced in cells treated with t10,c12-CLA. Interestingly, while LPS exposure increased AEA levels in cells treated with OA, this rise was not observed in cells treated with either CLA isomer, as depicted in Figure 9A.

Cells treated with the c9,t11-CLA isomer demonstrated a marked extracellular release of AEA, as illustrated in Figure 6E. Both POEA and OEA were also released into the media from c9,t11-CLA-treated cells, although this release was diminished by LPS exposure (Figures 9F, G). Conversely, docosahexaenoylethanolamide (DHAEA) levels, originating from docosahexaenoic acid (DHA), remained stable within the cells (Figure 9D). However, the extracellular release of DHAEA was significantly more elevated, showing increases of 2.3- and 3.8-fold in media from cells treated with c9,t11-CLA and t10,c12-CLA, respectively. This release was further augmented after LPS treatment, which led to increases of 2.8- and 4.3-fold, correspondingly (Figure 9H).

## 4 Discussion

Neuroinflammation represents a common feature in most neurodegenerative diseases and is characterized by activation of glial cells, release of inflammatory mediators, such as cytokines and chemokines, and generation of reactive oxygen and nitrogen species (Kempuraj et al., 2016). The main mediators of neuroinflammation are microglia cells, the resident immune cells of the CNS. Microglial activation occurs in response to changes in homeostasis and pathological insults to the brain. Activated microglia rapidly change their morphology and expression of diverse molecules inducing neuronal damage and dysfunction and promoting the progression of neurodegeneration (Colonna and Butovsky, 2017). Hence, inhibiting the persistent activation of microglia and mitigating the release of pro-inflammatory molecules emerges as a potentially effective strategy for both the prevention and treatment of neurodegenerative diseases.

The present study provides compelling evidence for the modulatory impact of CLA isomers on BV-2 microglial cell activation. In fact, *in vitro* incubation with both c9,t11-CLA and t10,c12-CLA isomers demonstrated a significant reduction of the expression of cytokines and other inflammatory mediators in BV-2 microglial cells, both under physiological conditions and after LPS stimulation. CLA has been reported to exhibit anti-inflammatory and immunomodulatory effects in animal models (Yang et al., 2015) and humans (Haghighatdoost and Nobakht M. Gh, 2018; Rastgoo et al., 2023). Numerous studies have reported the ability of CLA to reduce secretion of pro-inflammatory cytokines in different cell types, such as macrophages (Yu et al., 2002), bronchial epithelial cells (Huang et al., 2016), and peripheral blood mononuclear cells (Kim et al., 2011a). CLA can cross the BBB, reaching the CNS where is incorporated and metabolized (Fa et al., 2005) and exerts various neuroprotective functions, including protection from glutamate excitotoxicity (Hunt et al., 2010), memory improvement (Gama et al., 2015), and prevention of cognitive decline in animal models of neurodegenerative diseases (Monaco et al., 2018; Binyamin et al., 2019). Recent evidence further suggests CLA's potential role in demyelinating disorders by regulating autophagic lysosomal overactivation and cellular dysfunction in microglia (Zhou et al., 2023).

BV-2 cells represent a well-characterized and widely used model system for microglia. In this study, after treatment with LPS to establish a neuroinflammation cell model, most of cells acquired an activated phenotype and increased the expression of *CCL5, IL-1*β, *IL-6*, and *iNOS*. CLA isomers prevented BV-2 activation and reduced the expression of those inflammatory mediators, all involved in neurodegenerative processes. For example, CCL5 is a chemokine involved in several neurodegenerative diseases, neuroinflammation, and excitotoxic neurodegeneration (Cartier et al., 2005; Musante et al., 2008). Moreover, IL-1β may induce synaptic loss and contribute to neuronal injury and cell death (Allan et al., 2005; Sheppard et al., 2019).

In our study, IL-1β was undetectable in the cell culture supernatant of LPS-activated BV-2 cells; however, the intracellular precursor, pro-IL-1β, was identified. This observation aligns with the understanding that IL-1β maturation and secretion are rigorously regulated processes, mediated by cytosolic inflammasome complexes. These complexes activate caspase-1, which is essential for the cleavage of pro-IL-1β into its active form (Chan and Schroder, 2019). Our findings suggest that, in BV-2 cells, LPS stimulation alone does not suffice to trigger the NLRP3 inflammasome activation, indicating that additional stimuli are necessary for this process (Fan et al., 2017).

In the present study, we observed that CLA isomers can suppress the expression of inflammatory molecules in both LPS-activated and unstimulated microglial cells. Notably, BV-2 microglia, when cultured in serum-supplemented media, exhibit minimal activation levels, as demonstrated in our study (shown in Figure 2) and supported by findings from Laurenzi et al. (2001). This minimal activation state mirrors the low-grade inflammation characteristic of the aging brain, which contributes to increased risks of cognitive decline and heightened vulnerability to neurodegenerative diseases (Kempuraj et al., 2016; Matt and Johnson, 2016). Our findings suggest that CLA has the potential to mitigate the risk of neurodegenerative conditions in older adults by downregulating pro-inflammatory mediators associated with low-grade neuroinflammation.

These results are consistent with previous research demonstrating the neuroprotective properties of dietary CLA in a mouse model of age-related neurodegeneration, which suggested that CLA prompts an adaptive response beneficial for brain health (Monaco et al., 2018). Accordingly, we have reported that dietary CLA intake was associated with reduced inflammatory markers in cerebrospinal fluid and enhanced somatosensory evoked potentials in female carriers of X-linked adrenoleukodystrophy (Cappa et al., 2012). Furthermore, a comparative study revealed elevated levels of CLA and DHAEA in elderly individuals residing in a High-Longevity Zone in Sardinia's east-central mountainous region compared to younger individuals and elderly residents of a Low-Longevity Zone (Manca et al., 2021), suggesting a link between these compounds and longevity.

The mechanisms underlying effects of CLA remain largely unexplored and necessitate further investigation. The present study suggests three possible molecular mechanisms of action that may synergistically contribute to the anti-inflammatory effect of CLA.

(1) The increased *ACOX1* expression paralleled the β-oxidation of both CLA isomers within peroxisomes in BV-2 microglial cells, yielding CD16:2 metabolite. ACOX1 enzyme catalyzes the first and rate-limiting step of peroxisomal FA β-oxidation and participates in maintaining immune homeostasis in the organism. ACOX1 is widely expressed in the brain (Farioli-Vecchioli et al., 2001) and its deficiency causes a severe neuroinflammatory and neurodegenerative peroxisomal disease, pseudoneonatal adrenoleukodystrophy (Poll-The et al., 1988). LPS induces a decrease in *ACOX1* mRNA expression and consequently peroxisomal dysfunction and lipid metabolism alteration (Vamecq et al., 2018). A peroxisomal defect induces microglia cells to acquire a reactive phenotype associated with the upregulation of pro-inflammatory molecules (Tawbeh et al., 2023). The anti-inflammatory role of ACOX1 is mediated by inactivation of oxygenated eicosanoids via peroxisomal β-oxidation which also generates precursors of potent anti-inflammatory mediators, such as resolvins (Di Cara et al., 2019). We observed that CLA not only prevented LPS-induced reduction of the ACOX1 activity in BV-2 cells exposed to LPS, but also induced an increase in *ACOX1* expression in unstimulated BV-2 cells. The up-regulation of ACOX1 in mouse livers following dietary CLA intake has been described by Belury et al. (1997) probably mediated via peroxisome proliferator-activated receptor-α (PPAR-α) activation. Our data revealed that the CD16:2 β-oxidized metabolite, derived from c9,t11-CLA was promptly released into the medium. Further studies should be made to establish whether the release of CD16:2 into the medium might possess neuroprotective role as a ligand for PPAR alpha in other neural cells such as oligodendrocytes, astrocytes and neurons. It has been shown that c9,t11-CLA promotes proliferation of rat neural progenitor cells, while the isomer t10,c12-CLA, whose CD16:2 metabolite is not released in the medium in our experimental model, had the opposite effect (Wang et al., 2011).

(2) Importantly, t10,c12-CLA isomer exhibited distinct effect, decreasing MUFAs levels, as indicated by a 66% decrease in the POA/PA ratio by a reduction of SCD1 activity. These findings are consistent with a previous study suggesting that FAs with c12 double bond appear to be a crucial structural feature for inhibiting SCD1 activity, especially when paired with a t10 double, whereas a c11 double bond is less effective (Ntambi and Miyazaki, 2004). SCD1, a key enzyme regulator of lipid metabolism and MUFAs synthesis, appears to exert a dual influence on the microglia phenotype depending on the disease context. A recent study has shown that MUFAs generated by SCD1 can drive microglia and macrophages toward an inflammatory phenotype, influencing inflammatory and repair processes. SCD1 inhibition led to an anti-inflammatory shift in macrophages and microglia, enhancing remyelination (Bogie et al., 2020). We therefore suggest that the potential anti-neuroinflammation role of t10,c12-CLA might be at least in part exerted by SCD1 inhibition.

(3) Both CLA isomers have been demonstrated to significantly alter FA metabolism and the profile of FA-derived lipid mediators, such as NAEs, which play a pivotal role in modulating neuroinflammation (Saba et al., 2019; Murru et al., 2021a), In our current investigation, exposure to both CLA isomers resulted in a reduction of AEA levels in BV-2 microglial cells treated with LPS. Notably, the isomer t10,c12-CLA was also associated with decreased levels of POEA and OEA, potentially due to diminished availability of their MUFA precursors, POA and OA. Conversely, incubation with c9,t11-CLA markedly elevated the release of AEA, POEA, and OEA into the medium, an effect negated by LPS exposure. The suppression of OEA release by LPS could hinder OEA's role in modulating microglial phenotype transition from the pro-inflammatory M1 to the anti-inflammatory M2 state, a process mediated by PPAR alpha (Li et al., 2023).

Interestingly, while CLA isomers did not alter intracellular levels of DHAEA, derived from DHA, in BV-2 cells, they prompted its release into the medium, particularly observed with t10,c12-CLA in both untreated and LPS-stimulated conditions.

DHAEA, also known as synaptamide, may significantly contribute to the anti-neuroinflammatory activity of CLA. *In vivo* studies have demonstrated DHAEA's capacity to mitigate pro-inflammatory cytokine production, including the suppression of LPS-induced microglial activation and pro-inflammatory cytokine expression (Park et al., 2016). Prior research indicates that DHAEA activates GPR110 receptor, inducing upregulation of cAMP in CNS microglia and peripheral macrophages. cAMP in turn may inhibit NF-κB transcriptional activity, thereby reducing microglial activation and pro-inflammatory cytokine output (Park et al., 2019). Furthermore, DHAEA has been recognized for its biological activity that promotes neurogenesis and synaptogenesis (Kim et al., 2011b; Rashid et al., 2013; Kharebava et al., 2015; Kim and Spector, 2018).

The use of an *in vitro* model involving a single cell type, such as the BV-2 microglial cell line, inherently presents certain limitations, primarily due to the absence of interactions with other cell types, including neurons and other glial cells. Additionally, the BV-2 cell line exhibits reduced reactivity compared to primary microglial cells when stimulated with LPS. Nevertheless, BV-2 cells retain several key microglial characteristics, including phagocytic activity and the ability to secrete NO and pro-inflammatory cytokines upon stimulation (Henn et al., 2009). These features render BV-2 cells highly suitable for biochemical and molecular studies that require large cell quantities, and they remain the most widely employed cell line for investigating microglial behavior and inflammatory responses *in vitro*. Given the critical role of microglia in neuroinflammation, a comprehensive understanding of their behavior is essential for elucidating the mechanisms by which CLA isomers influence microglial activity. In this context, *in vitro* models are indispensable tools for generating foundational knowledge.

In conclusion, our study implies that the anti-inflammatory effects demonstrated by CLA isomers may be attributed to their distinct impact on FA metabolism and modulation of bioactive FA-derived NAEs. This insight offers a promising avenue for therapeutic intervention aimed at preventing disorders where neuroinflammation plays a central role. By addressing neuroinflammation, particularly by mitigating microglial activation and increasing the release of anti-inflammatory mediators, a potential strategy emerges to offer an environment conducive to neuronal function and homeostasis. This underscores the potential of targeting fatty acid metabolism as a viable therapeutic approach to counteract neuroinflammation-associated disorders.

Despite the acknowledged limitations of *in vitro* systems, our findings provide a basis for future research that includes studies on additional brain cell types and *in vivo* experiments. These subsequent studies will be crucial for confirming the observed mechanisms *in vivo* and further exploring the anti-inflammatory properties of CLA.

## Data Availability

The raw data supporting the conclusions of this article will be made available by the authors, without undue reservation.
