# Supplementary material for: Anti-neuroinflammatory effects of conjugated linoleic acid isomers, c9,t11 and t10,c12, on activated BV-2 microglial cells
Source: Front Cell Neurosci. 2024 Sep 27;18:1442786. doi: 10.3389/fncel.2024.1442786 (PMC11466893; doi:10.3389/fncel.2024.1442786)
Supplement: Supplementary file 1 [file Data_Sheet_1.PDF]

Supplementary Material

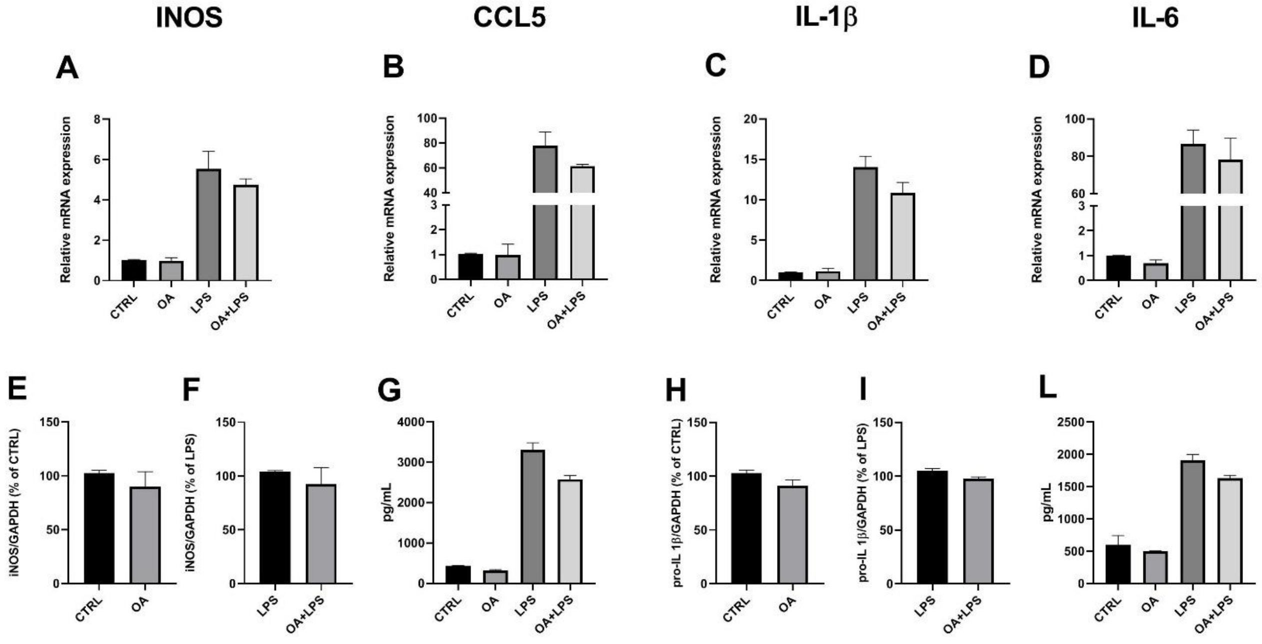

Supplementary Figure 1: Impact of oleic acid (OA) treatment on the expression of pro-inflammatory markers in unstimulated and LPS-stimulated BV-2 cells.
mRNA expression of iNOS (A), CCL5 (B), IL-1β (C), and IL-6 (D) was assessed using real-time PCR. The secretion of CCL5 (G) and IL-6 (L) was evaluated by ELISA. Intracellular levels of iNOS (E-F) and pro-IL-1β (H-I) were measured by western blot.
No significant differences were observed between OA treated and untreated BV-2 cells.
